# Supplementary material for: A low-power vertical dual-gate neurotransistor with short-term memory for high energy-efficient neuromorphic computing
Source: Nat Commun. 2023 Oct 11;14:6385. doi: 10.1038/s41467-023-42172-y (PMC10567726; doi:10.1038/s41467-023-42172-y)
Supplement: Supplementary file 1 — Supplementary Information [file 41467_2023_42172_MOESM1_ESM.docx]

**Supplementary materials**

**A low-power vertical dual-gate neurotransistor with short-term memory for high energy-efficient neuromorphic computing**

Han Xu^1,2,3,4^, Dashan Shang*^1,2,3^, Qing Luo^1,2,3^, Junjie An^1,2^, Yue Li^1,2,3^, Shuyu Wu^1,2,3^, Zhihong Yao^1,2^, Woyu Zhang^1,2,3^, Xiaoxin Xu^1,2,3^, Chunmeng Dou^1,2,3^, Hao Jiang^5^, Liyang Pan^4^, Xumeng Zhang^5^, Ming Wang^5^, Zhongrui Wang^6^, Jianshi Tang*^4^, Qi Liu*^1,2,5^, Ming Liu^1,2,5^

^1^State Key Lab of Fabrication Technologies for Integrated Circuits, Institute of Microelectronics, Chinese Academy of Sciences, Beijing 100049, China

^2^Key Laboratory of Microelectronics Devices and Integrated Technology, Institute of Microelectronics, Chinese Academy of Sciences, Beijing 100049, China

^3^University of Chinese Academy of Sciences, Beijing 100049, China

^4^School of Integrated Circuits, Beijing National Research Center for Information Science and Technology (BNRist), Tsinghua University, Beijing, China

^5^Frontier Institute of Chip and System, Fudan University, Shanghai 200433, China

^6^Department of Electrical and Electronic Engineering, The University of Hong Kong, Hong Kong 999077, Hong Kong

*Corresponding author. E-mail: [shangdashan@ime.ac.cn](mailto:shangdashan@ime.ac.cn,); jtang@tsinghua.edu.cn; qi_liu@fudan.edu.cn

This **Supplementary Information** contains:

**Figure S1.** Implementation of ultrashort channel EGT.

**Figure S2.** Feasibility of large-area fabrication using the vertical structure constructing method.

**Figure S3.** A two-layer V-EGT.

**Figure S4.** Elemental mapping of V-EGT.

**Figure S5.** Mechanism of EGT.

**Figure S6.** DC electrical characterization of V-EGT.

**Figure S7.** The discharge circuit and corresponding equivalent circuit model of EGT.

**Figure S8.** Variable channel length characteristics of V-EGT.

**Figure S9.** Symmetry of the source-drain electrodes of V-EGT.

**Figure S10.** Dilemma of read voltage and read power in planar EGT.

**Figure S11.** Read voltage comparison among various EGTs.

**Figure S12.** Characteristics of V-EGT with variable channel width.

**Figure S13.** fW-level read power of V-EGT.

**Figure S14.** Synergistic scalability of the horizontal and vertical dimensions of EGT.

**Figure S15.** Low voltage operation of V-EGT.

**Figure S16.** Consistency between different gates of dual-gate V-EGT.

**Figure S17.** Current-time curves of dual-gate V-EGT in response to different gate pulse a) amplitudes and b) widths.

**Figure S18.** Relationship between t and ITD.

**Figure S19.** Simulation results of applying voltage to V-EGT by voltage divider circuit.

**Table S1.** Comparison of V-EGTs in material type, device fabrication, key device parameters, device performance, targeted biomimetic functionality, and demonstrated applications.

**Table S2.** Estimated read time and read energy consumption for various EGTs.

**Table S3.** Comparison among various EGTs in terms of write energy.

**Note 1.** Discussion on the Use of Write-Read Delay of LTM EGTs to Calculate Their Read Energy Consumption

**Note 2.** Discussion on the Decay Time of STM EGT

**Note 3.** Estimation of V-EGT Device Footprint in Array Configuration

**Note 4.** Realizing the Analog Computing Characteristics of Neuron's Coincidence Detection on Dual-Gate V-EGT

**Note 5.** The Principle of Sound Azimuth Recognition Ability of the Dual-Gate V-EGT-Based Neural Network

**Note 6.** The Principle of Sound Distance Recognition Capability in Our Dual-Gate V-EGT-Based Neural Network


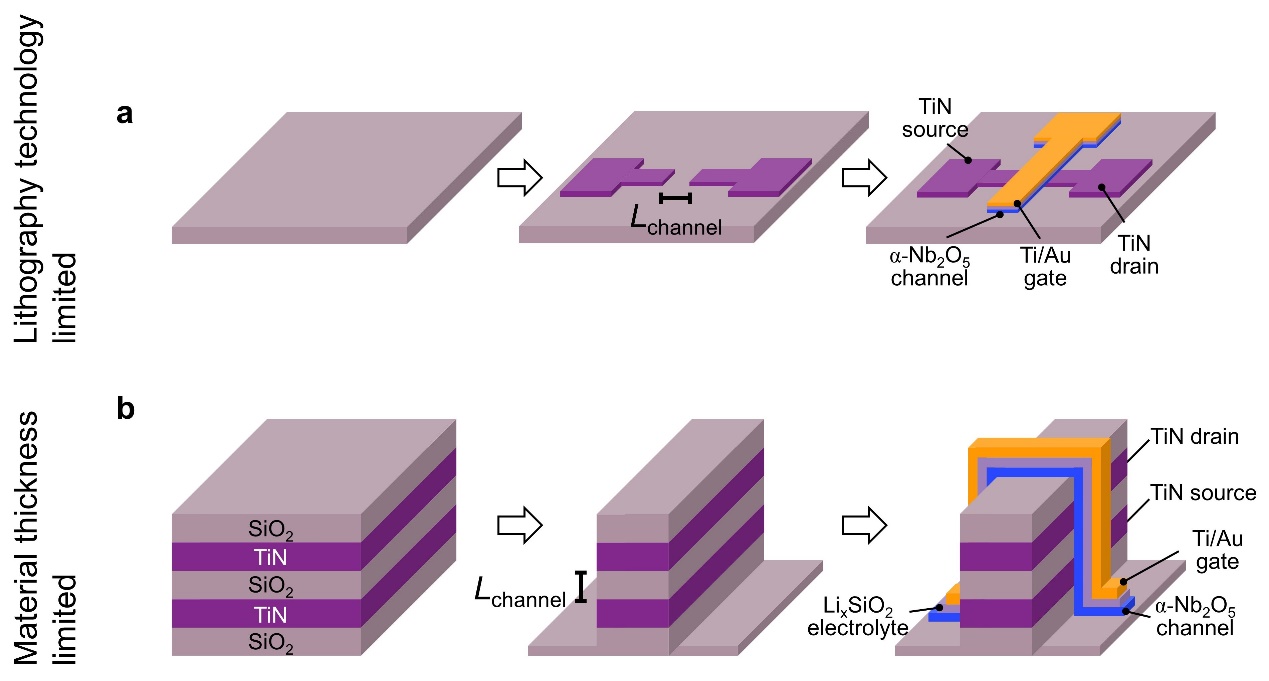


**Supplementary Figure S1. Implementation of ultrashort channel EGT. a)** Planar EGT manufacturing process: substrate cleaning, deposition of source/drain electrodes, deposition of channel/electrolyte/gate stack. **b)** V-EGT manufacturing process: deposition of electrode/spacer/electrode/spacer stack, one-step etch to substrate, deposition of channel/electrolyte/gate stack. Purple represents source/drain electrodes, gray represents spacer or electrolyte or substrate, blue represents the channel, and yellow represents the gate. The ultra-short channel can be achieved using the vertical structure. The fabrication complexity of EGT is not significantly increased according to the V-EGT construction method presented here.

V-EGTs indeed require more material consumption compared to planar EGTs. Planar EGTs consist of four layers, that is, source/drain electrode, channel, electrolyte, and gate, while the V-EGTs involve seven layers, that is, electrode/insulator/electrode/insulator stack (specifically TiN/SiO_2_/TiN/SiO_2_), channel, electrolyte, and gate. In the V-EGT design, it is worth noting that the topmost layer of SiO_2_ in the TiN/SiO_2_/TiN/SiO_2_ stack can be omitted (similar to the approach adopted by Duan *et al*. in vertical IGZO TFTs^1^), reducing the materials consumption to six layers. Although V-EGTs require more materials consumption, they can achieve ultrashort channel lengths in a simple and cost-effective manner, whereas planar EGTs would require expensive lithography equipment (such as electron beam lithography) to achieve sub-100-nanometer channel lengths.


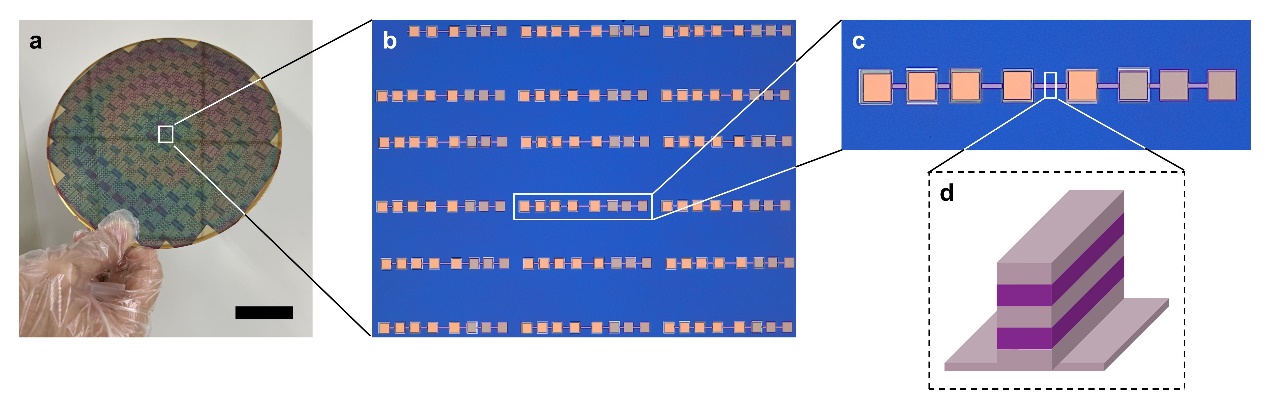


**Supplementary Fig. S2. Feasibility of large-area fabrication using the vertical structure constructing method.** **a)** Optical microscopy (OM) image of an 8-inch wafer-scale fabrication of the dual-gate V-EGT. **b) - d)** Magnified views of (a) at different magnification levels.


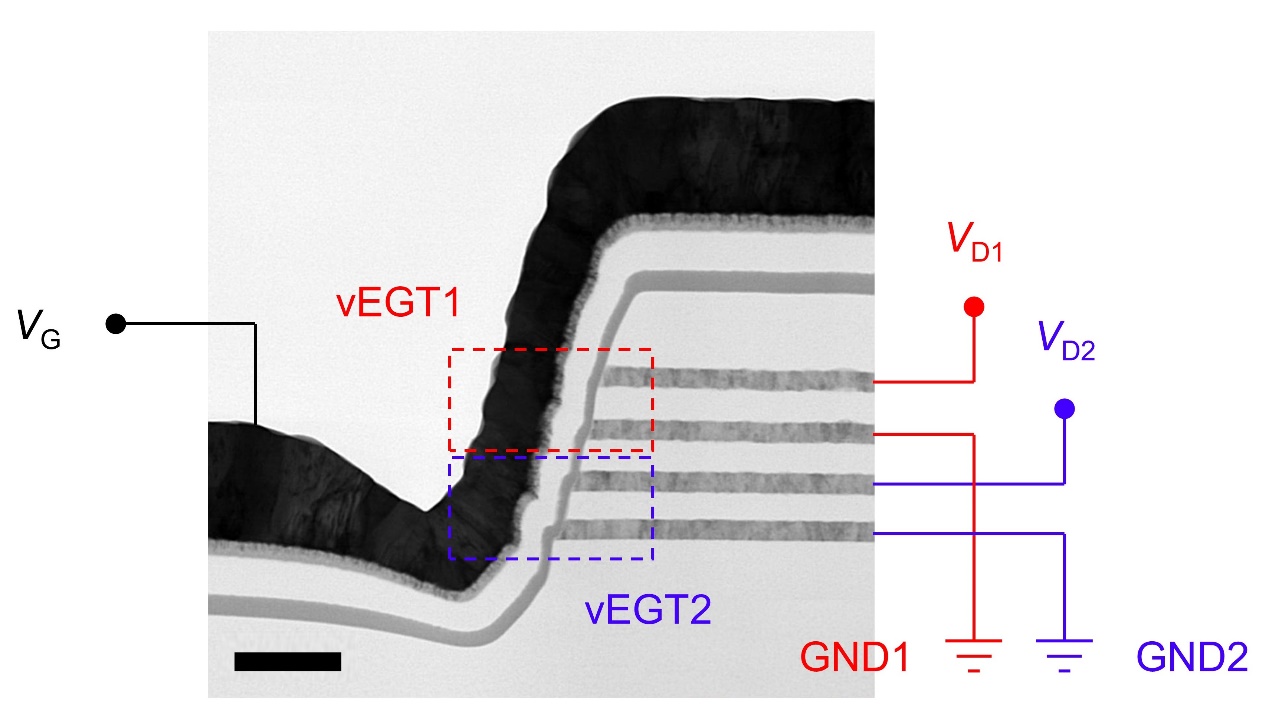


**Supplementary Fig. S3. A two-layer V-EGT.** The image shows a cross-sectional view of a two-layer V-EGT, with two V-EGTs marked respectively with red and blue dashed boxes. The scale bar is 100 nm.


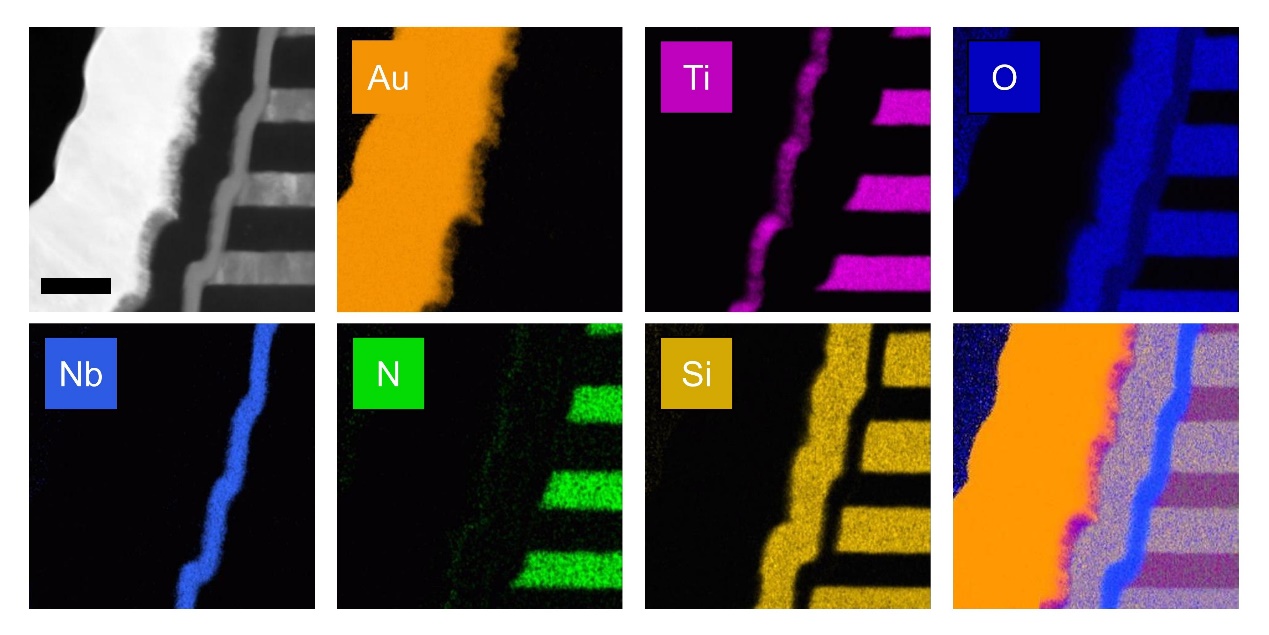


**Supplementary Fig. S4. Elemental mapping of V-EGT.** Scale bar is 30 nm.


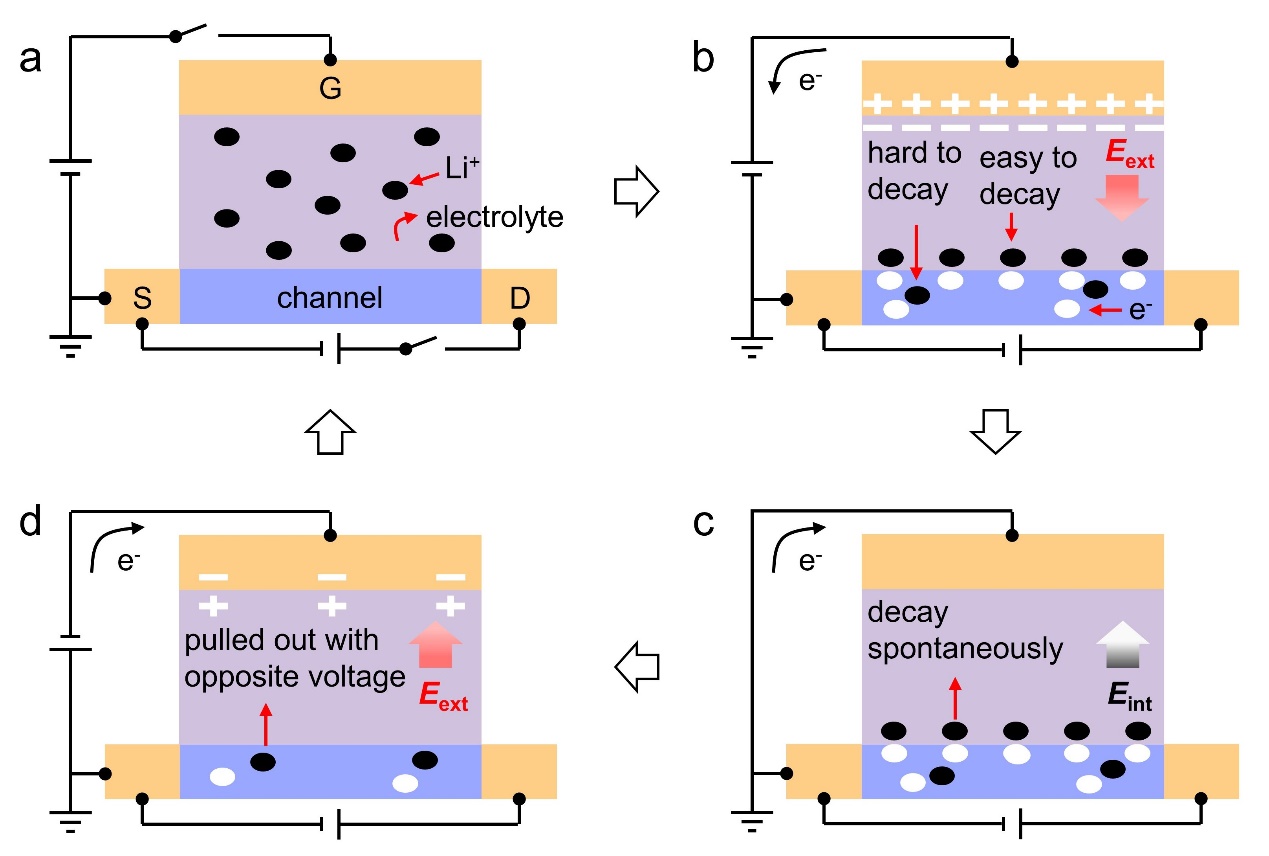


**Supplementary Fig. S5. Mechanism of EGT.** EGT exhibits a hybrid mechanism of electric double layer and ion intercalation/deintercalation^2^. The electric double layer mechanism arises from the accumulation of mobile electrolyte ions at the electrolyte/channel interface under the gate voltage. These ions diffuse back into the electrolyte after the voltage is removed, contributing to the STM (transient part) of the electrical properties of EGT. In the ion intercalation/deintercalation mechanism, the mobile ions in the electrolyte are injected into the channel under gate voltage and require a reverse voltage to remove them completely, contributing to the LTM (permanent part) of the electrical characteristics of EGT. At low excitation pulse intensities, the mobile ions inside the electrolyte lack sufficient energy to pass through the electrolyte/channel interface, leading to nearly complete STM electrical properties of the device. As the pulse excitation strength increases, a certain proportion of electrolyte ions can pass through the electrolyte/channel interface and enter into the channel, resulting in a hybrid mechanism of electric double layer and ion intercalation/deintercalation and a certain degree of LTM in the device.


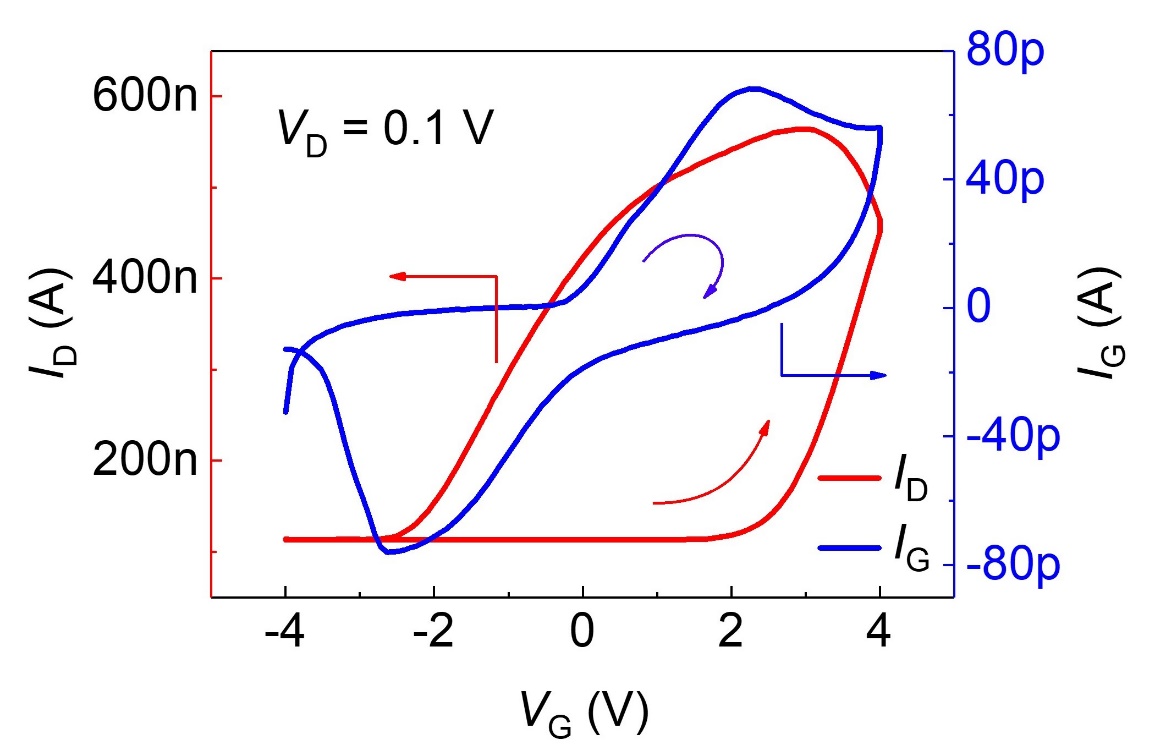


**Supplementary Fig. S6. DC electrical characterization of V-EGT.** Transfer characteristics and gate leakage of a V-EGT with a channel length of 30 nm.


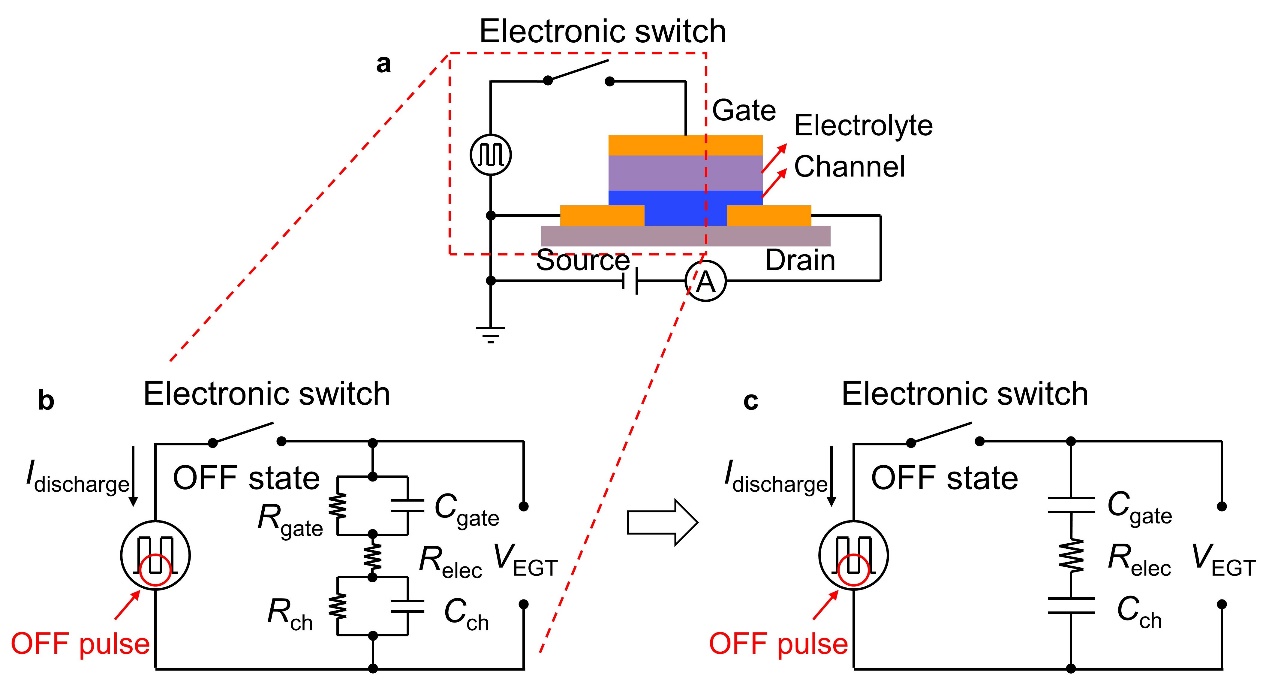


**Supplementary Fig. S7. The discharge circuit and corresponding equivalent circuit model of EGT.** **a)** Schematic diagram of the experimental setup for EGT. **b)** Equivalent circuit diagram of the gate-source write circuit of EGT (indicated by the red dashed box in **a**. **c)** Simplified equivalent circuit diagram of the gate-source write circuit of EGT.

**Supplementary Fig. S7a** shows the schematic diagram of the experimental setup for EGT. It involves applying a varying gate voltage at the gate of EGT, applying a constant DC bias voltage (such as 0.1 V) at the drain, and grounding the source. An electronic switch is connected in series between the gate of EGT and the pulse source.

**Supplementary Fig. S7b** presents the equivalent circuit diagram of the gate-source write circuit of EGT (indicated by the red dashed box in **Supplementary Fig. S7a**). In the circuit, *R*_gate_, *C*_gate_, *R*_elec_, *R*_ch_, *C*_ch_, and *V*_EGT_ represent the gate/electrolyte interface resistance, gate/electrolyte interface capacitance, electrolyte/channel resistance, electrolyte/channel capacitance, and the voltage across the gate and source of EGT, respectively. Considering that the leakage current at both the gate/electrolyte interface and the electrolyte/channel interface is very small, *R*_gate_ and *R*_ch_ can be neglected. Therefore, **Supplementary Fig. S7b** can be simplified to **Supplementary Fig. S7c**.

During the interval between two adjacent gate pulses (i.e., the OFF pulse), the previously charged *C*_gate_ and *C*_ch_ discharge in the opposite direction of charging, as indicated by the direction of the discharging current, *I*_discharge_. The retention of EGT can be quantified by the discharging time of the gate-source write circuit. Based on **Supplementary Fig. S7c**, the discharge time or speed of the gate-source write circuit can be expressed as *τ*_RC_ = *R* × *C*, where *R* includes the electrolyte resistance and the resistance of the electronic switch in series with the gate, and *C* contains *C*_gate_ and *C*_ch_.


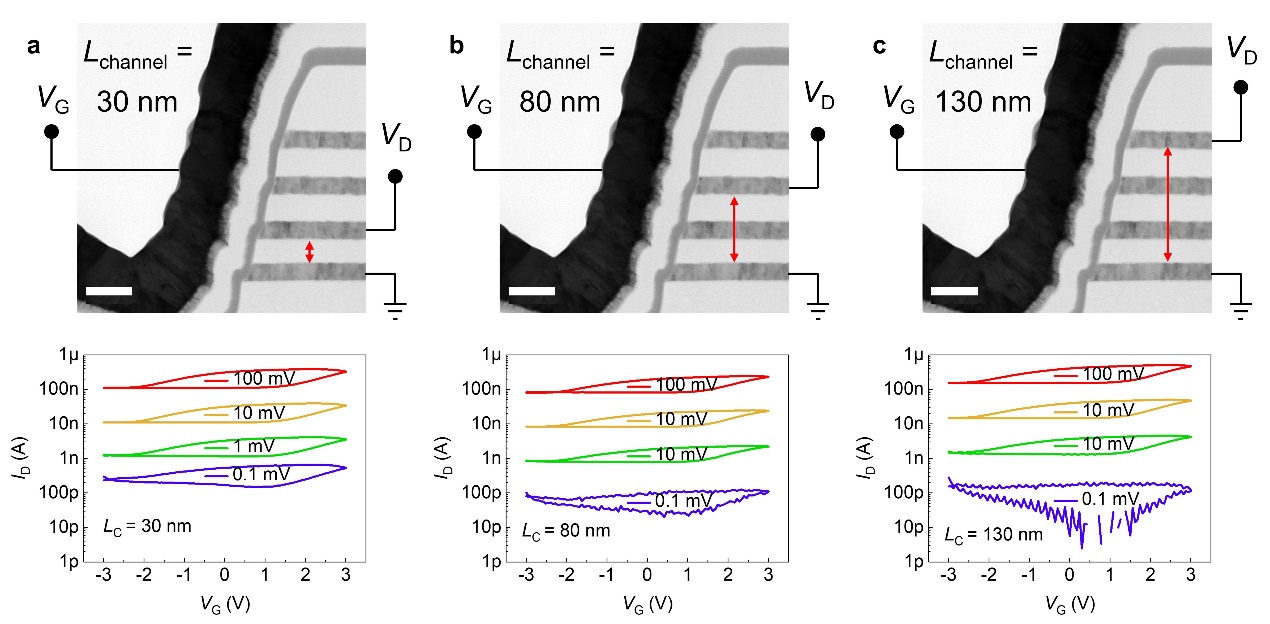


**Supplementary Fig. S8. Variable channel length characteristics of V-EGT.** **a) - c)** Transfer characteristic curves of V-EGT with different source-drain electrode selections at channel lengths of 30, 80, and 130 nm, respectively. The scale bar is 50 nm.


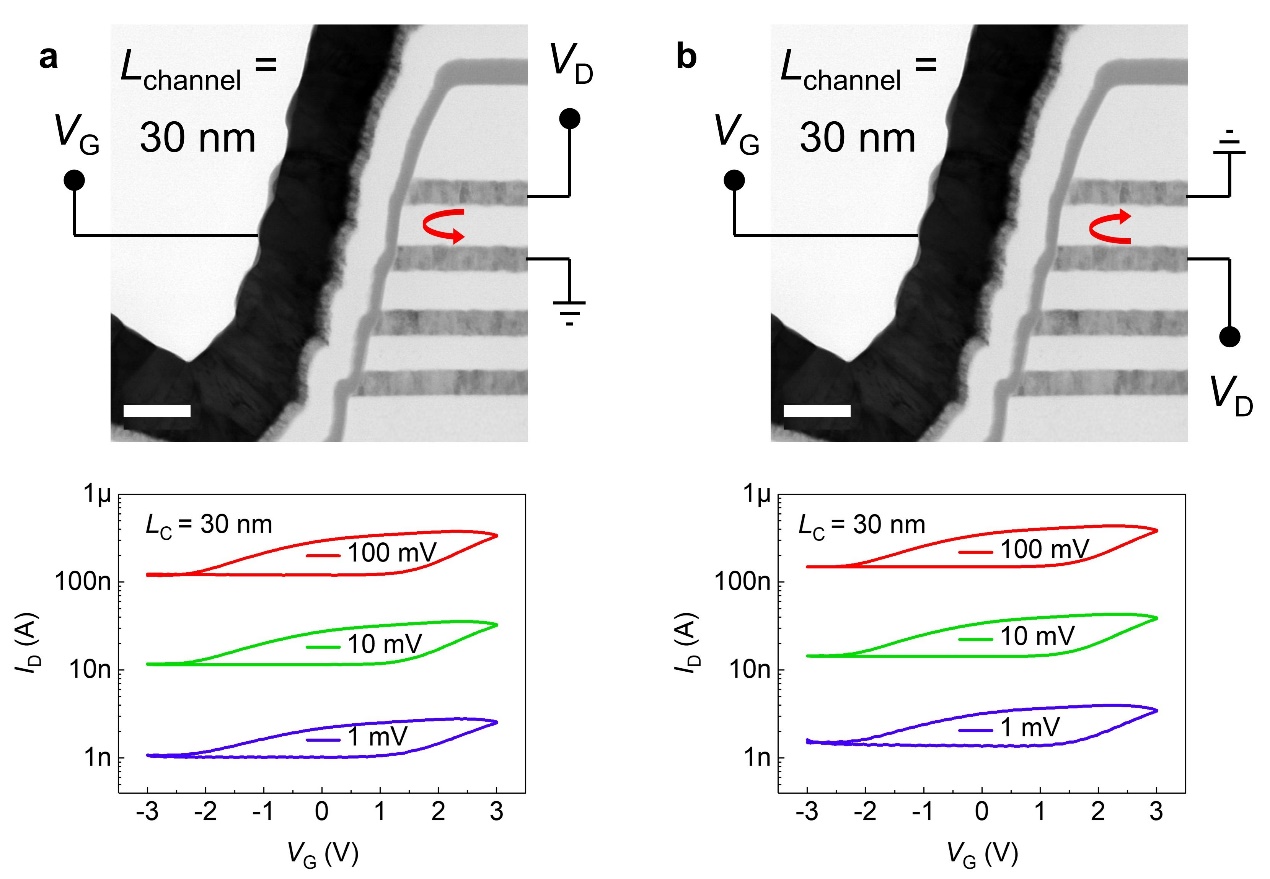


**Supplementary Fig. S9. Symmetry of the source-drain electrodes of V-EGT.** **a)** Transfer characteristic curve of the first selection scheme of source-drain electrode. **b)** Transfer characteristic curve of the second selection scheme of source-drain electrode.


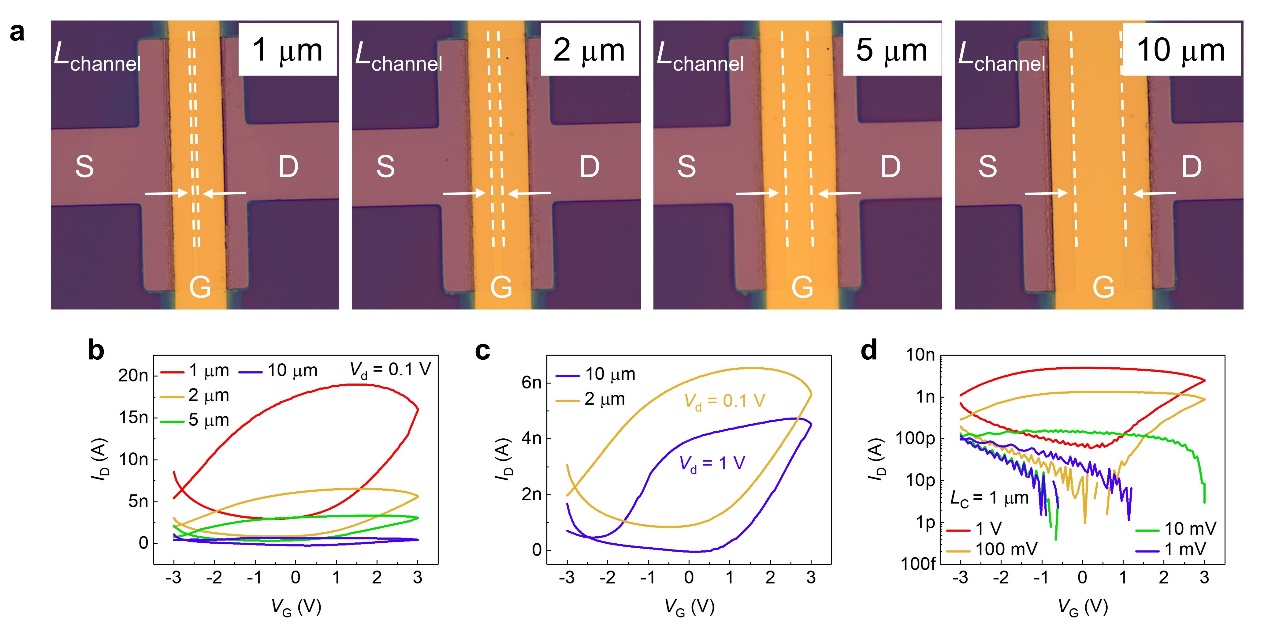


**Supplementary Fig. S10. Dilemma of read voltage and read power in planar EGT. a)** Optical micrographs of planar EGTs with different channel lengths. **b)** Transfer characteristics of planar EGTs with different channel lengths, read at 0.1 V. **c)** Transfer characteristics of planar EGTs with 2 µm and 10 µm channel lengths, read at 0.1 and 1 V, respectively. **d)** Transfer characteristics of planar EGT with 1 µm channel length at different read voltages.

Under the same channel current, a planar EGT with a longer channel requires a higher read voltage (Supplementary **Fig. 10c**), resulting in increased read power and read energy consumption. Scaling down the read voltage of planar EGTs with a 1 µm channel length below 10 mV is difficult (Supplementary **Fig. 10d**). This is due to the fact that, compared with short-channel V-EGTs, long-channel planar EGTs have a smaller intrinsic channel current (channel current with only read but no write voltage) at the same read voltage, making them more susceptible to gate leakage. Consequently, long-channel planar EGTs lose their gate control at a higher read voltage. Furthermore, the accuracy of the measurement instrument is typically limited to 1 pA. Therefore, long-channel planar EGTs are more likely to reach the lower current limit of the measurement instrument, which prevents the use of small read voltages.


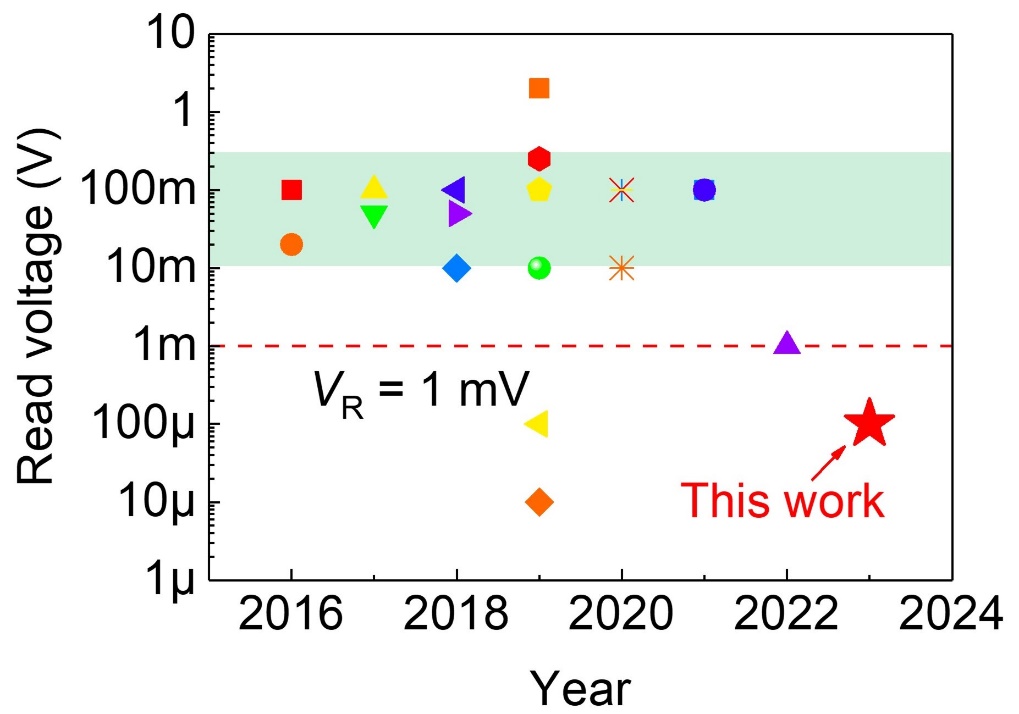


**Supplementary Fig. S11. Read voltage comparison among various EGTs.**


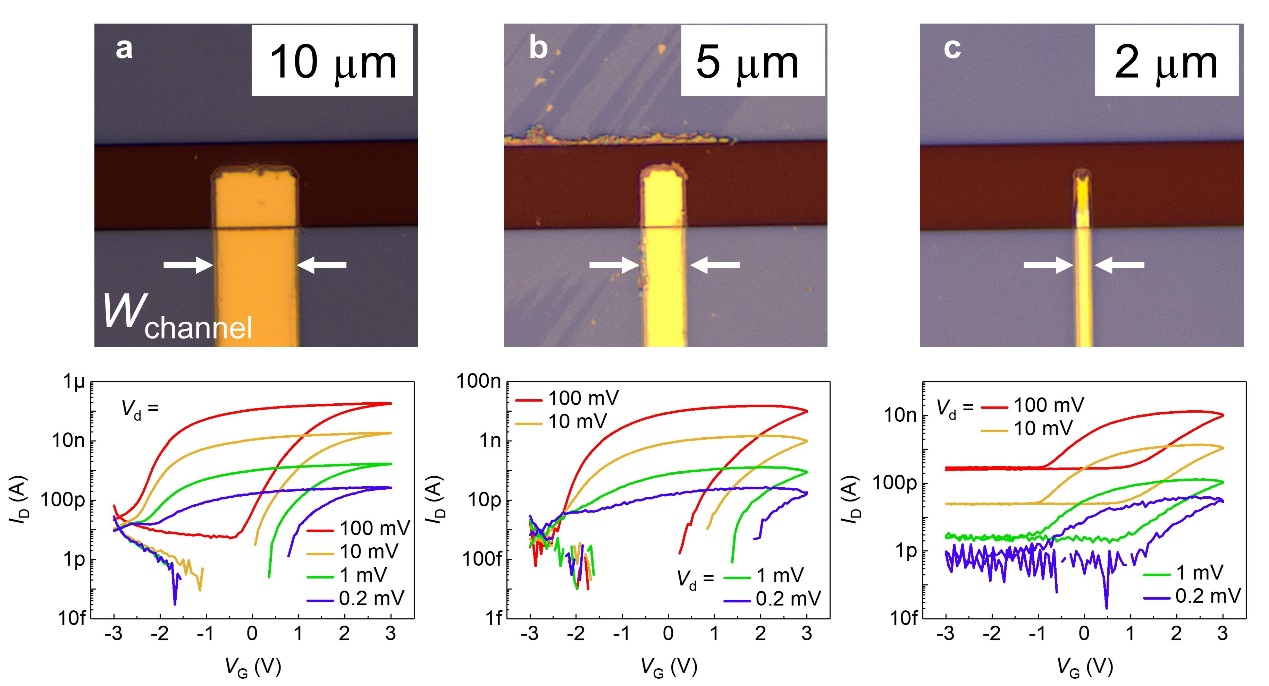


**Supplementary Fig. S12.** **Characteristics of V-EGT with variable channel width. a)** Optical micrograph of V-EGT with a channel width of 10 µm and the corresponding transfer characteristic curve. **b)** Optical micrograph of V-EGT with a channel width of 5 µm and the corresponding transfer characteristic curve. **c)** Optical micrograph of V-EGT with a channel width of 2 µm and the corresponding transfer characteristic curve.


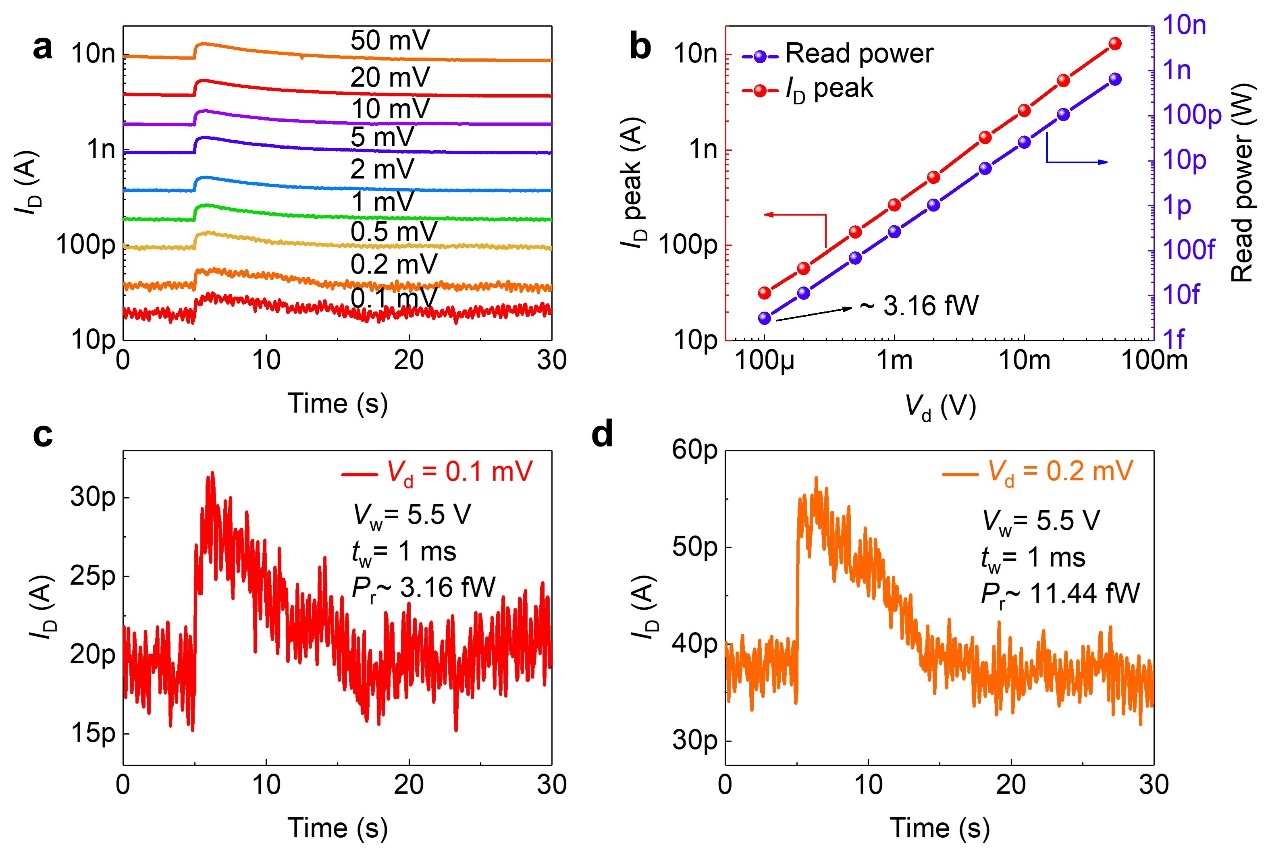


**Supplementary Fig. S13. fW-level read power of V-EGT. a)** Channel current-time response curves at different read voltages. The pulse amplitude is 5.5 V and the width is 1 ms. **b)** Relationship between the channel peak current and read power versus read voltage. **c)** and **d)** Channel current-time response curves at read voltages of 0.1 and 0.2 mV, respectively, with corresponding read powers of ~3.16 fW and ~11.44 fW, respectively.


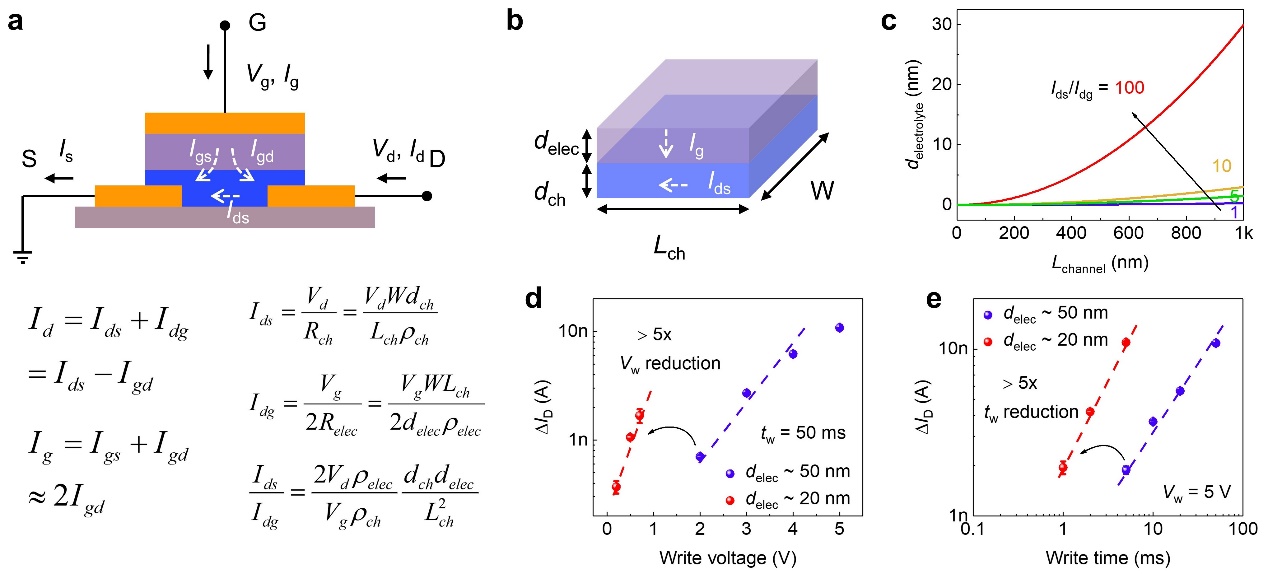


**Supplementary Fig. S14. Synergistic scalability of the horizontal and vertical dimensions of EGT. a)** Analysis of the current components of EGT and their relationship. **b)** The channel and electrolyte dimensions that determine the magnitude of the current in EGT. **c)** In order to obtain the same gating capability (quantified as *I*_ds_/*I*_dg_ here), the thickness of the electrolyte must decrease with decreasing channel length, specifically, *d*_elec_ is proportional to the square of *L*_ch_. **d)** and **e)** Improvement in write voltage and write speed of V-EGT compared to planar EGT.


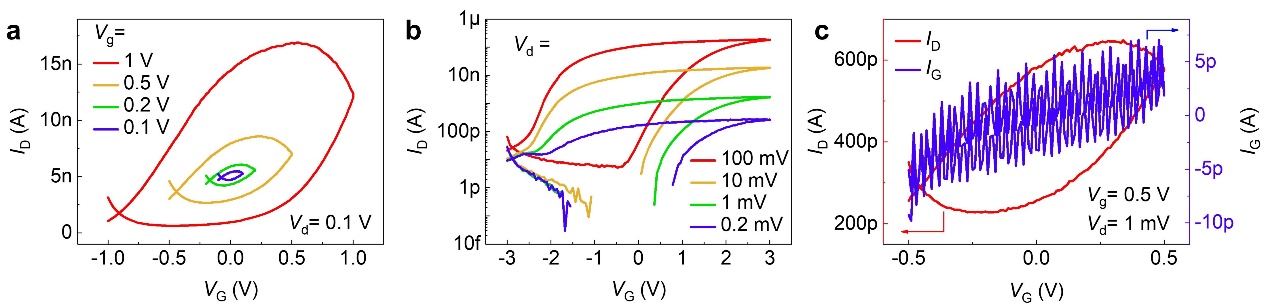


**Supplementary Fig. S15. Low voltage operation of V-EGT. a)** Reduction of the DC sweeping voltage range of V-EGT to 1 V and below. **b)** Reduction of the read voltage of V-EGT to 1 mV and below. **c)** Both low sweeping voltage range and low read voltage are achieved, with a sweeping voltage range of ±0.5 V and a read voltage of 1 mV.


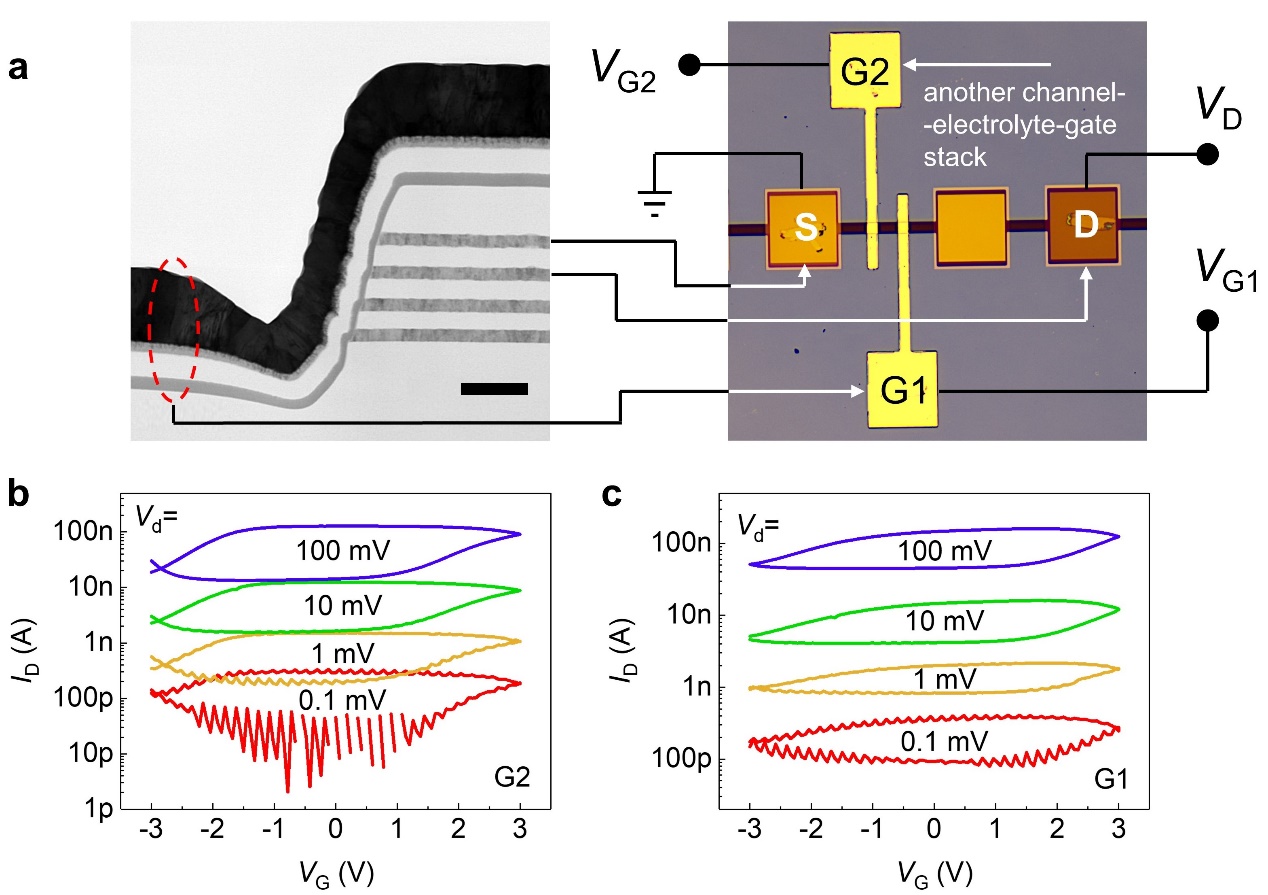


**Supplementary Fig. S16. Consistency between different gates of dual-gate V-EGT. a)** Optical micrograph of a dual-gate V-EGT with a channel length of 30 nm. **b)** Transfer characteristic curve of gate 1 operating independently. **c)** Transfer characteristic curve of gate 2 operating independently.

**
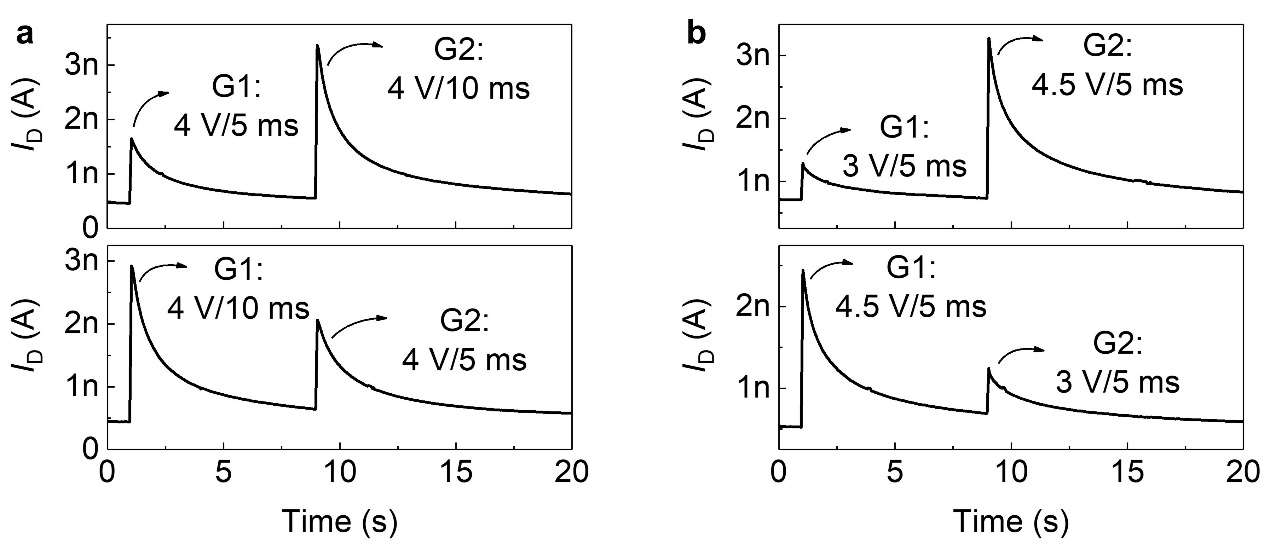
**

**Supplementary Fig. S17. Current-time curves of dual-gate V-EGT in response to different gate pulse a) amplitudes and b) widths.**


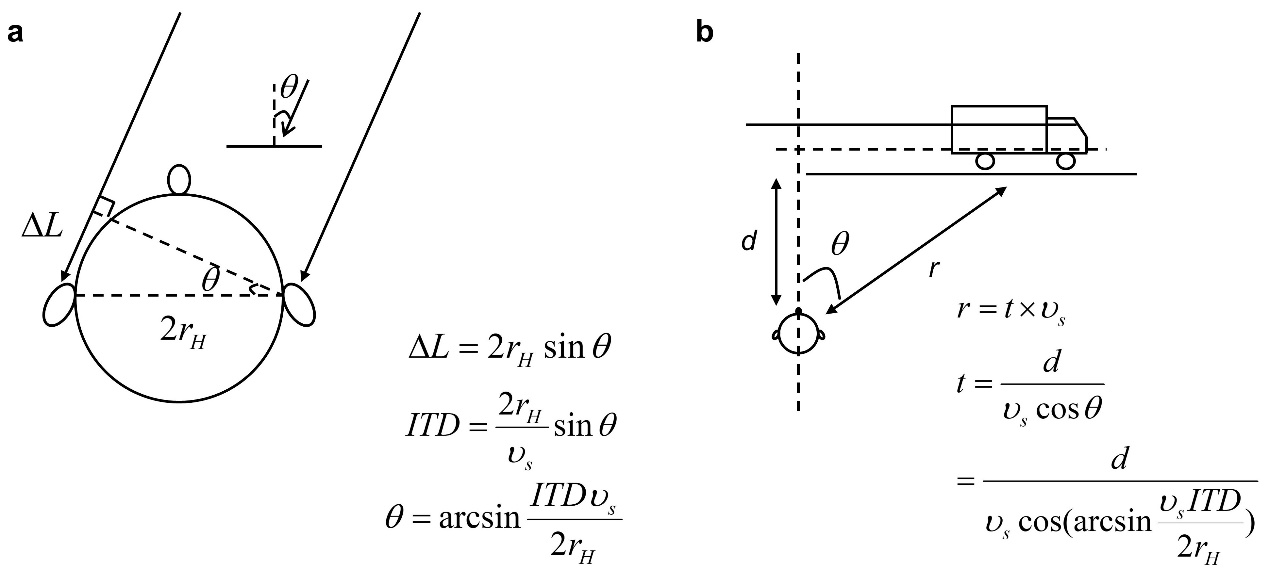


**Supplementary Fig. S18. Relationship between t and ITD.**


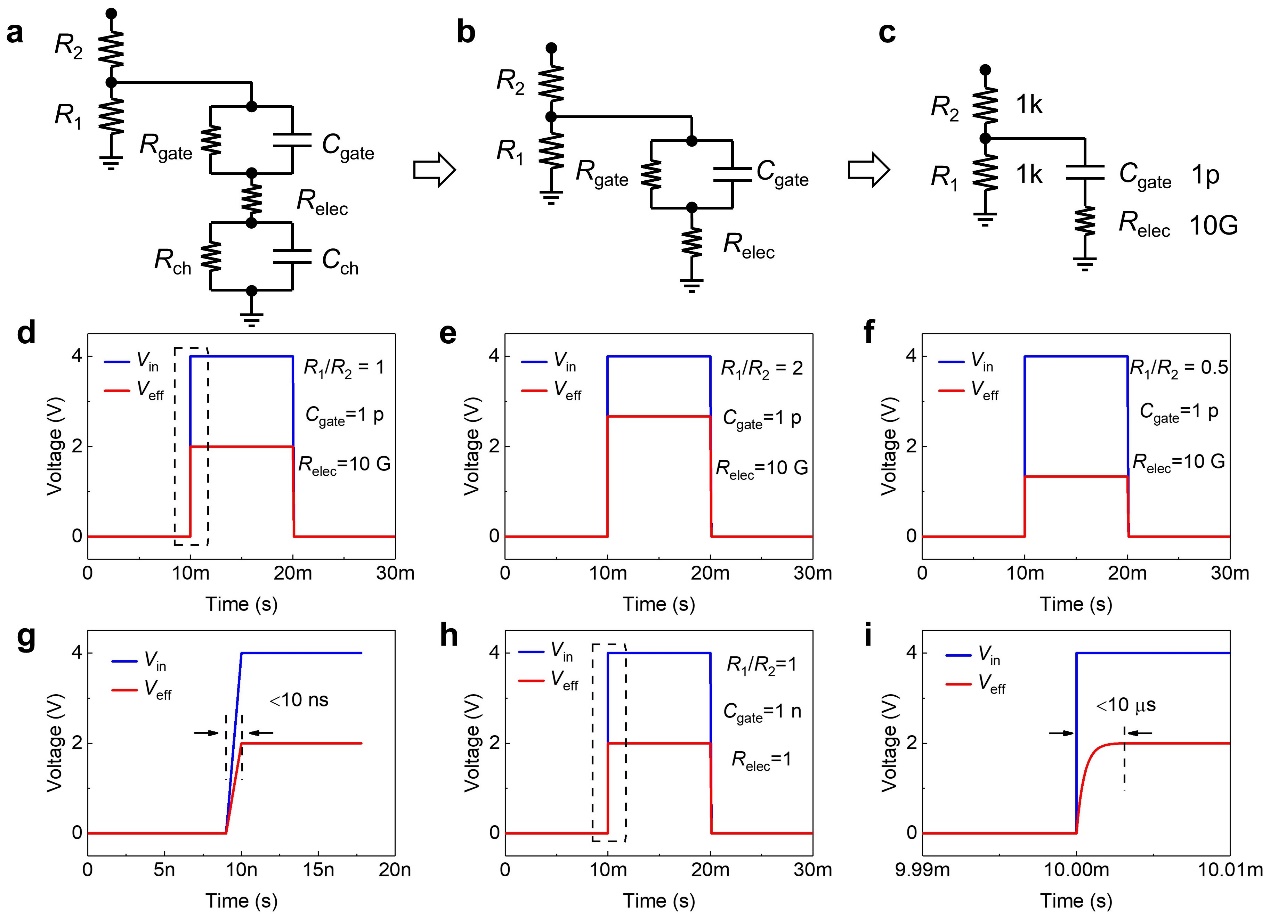


**Supplementary Fig. S19. Simulation results of applying voltage to V-EGT by voltage divider circuit. a)** Equivalent circuit diagram of applying voltage to V-EGT through a voltage divider circuit (gate-source circuit). After considering the actual resistances and capacitances of the EGT gate, electrolyte, and channel, as well as their effects on the voltage divider circuit, the circuit can be simplified as shown in **(b)** and **(c)**. For a V-EGT with a size of L × W = 30 nm × 10 µm, and based on the measured capacitance and the reported resistivity of the electrolyte, a reasonable value for the gate capacitance and electrolyte resistance has been given as 1 pF and 10 GΩ, respectively. **d) - f)** Input and output voltage waveforms of the voltage divider circuit when *R*_1_/*R*_2_ are 1, 2, and 0.5, respectively. In (d)-(f), *R*_1_ = 1 kΩ and *R*_2_ is 1 kΩ, 0.5 kΩ, and 2 kΩ, respectively. **g)** Magnified view of the position indicated by the box in (d) shows that, for the normal range of gate capacitance and electrolyte resistance, the voltage divider circuit can transmit the input voltage to the gate of V-EGT within 10 ns. **h)** and **i)** A larger gate capacitance and smaller electrolyte resistance will delay the transmission of the voltage divider circuit from the input voltage to the output voltage. Assuming a gate capacitance of 1 nF and an electrolyte resistance of 1 Ω, we simulate a situation that deviates from reality. Even so, the voltage divider circuit can still transmit the input voltage to the gate of EGT within 10 µs (i), much less than the width of the pulse (10 ms). Therefore, the proposed scheme of modulating the pulse amplitude through the voltage divider circuit (a new form of weight modulation on the pulse amplitude) is feasible.

**Supplementary Table T1. Comparison of V-EGTs in material type, device fabrication, key device parameters, device performance, targeted biomimetic functionality, and demonstrated applications^3-7^.**


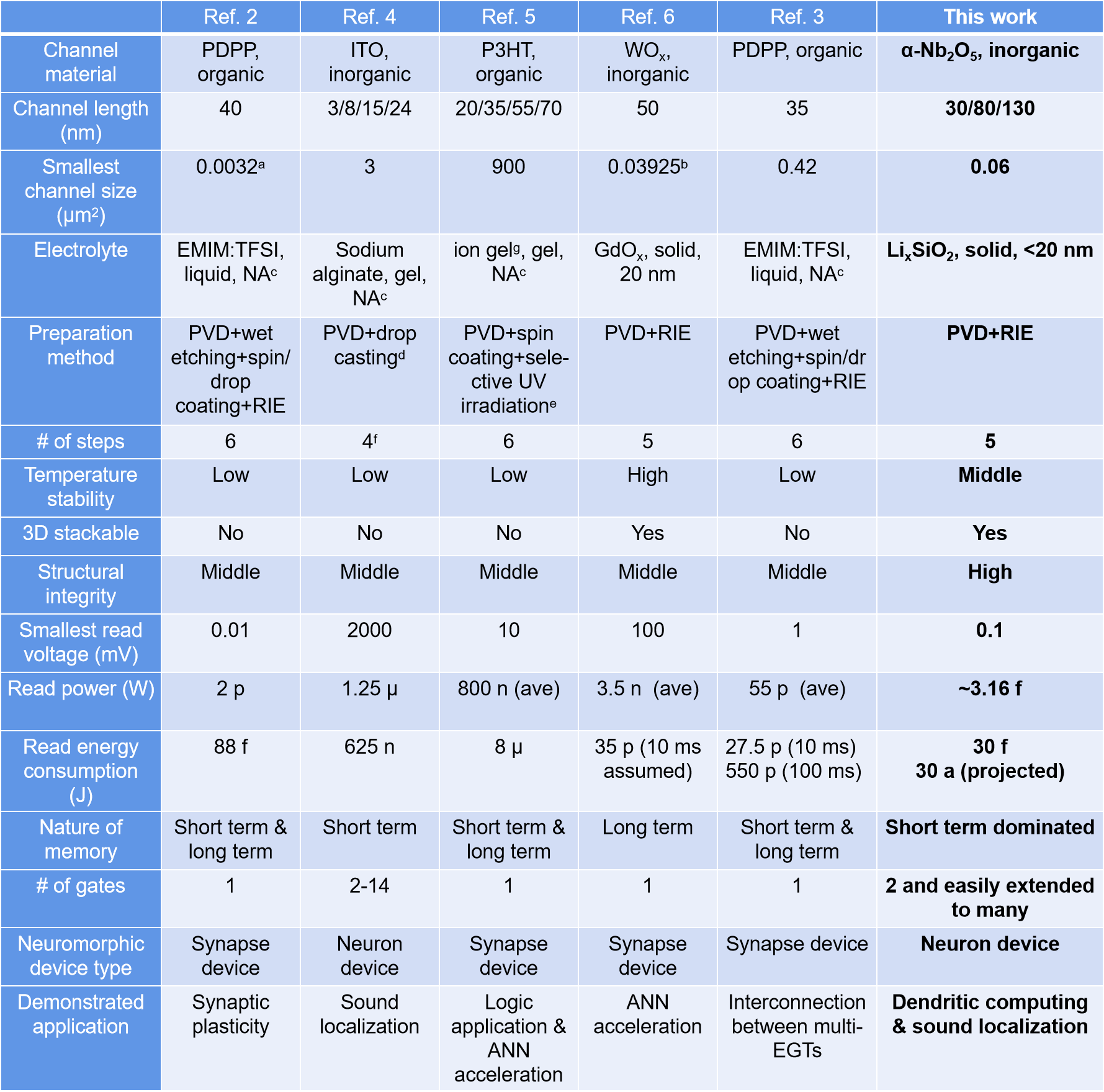


^a,b^Electron beam lithography was utilized for the fabrication of V-EGT in these two works. ^c^The thickness of the electrolyte is not provided in these works since either liquid or gel electrolytes were used. ^d,f^In this work, a hardmask was employed to fabricate the V-EGT. The hardmask technique generally results in simpler device fabrication steps but larger device size. ^e^Both hardmask and lithography methods were used to fabricate the V-EGT in this work. ^g^The ion gel was mixed with ionic liquid EMIM: TFSI and PVdF-HFP in this work. PDPP refers to diketopyrrolopyrrole-terthiophene donor-acceptor polymer, ITO refers to indium tin oxide, P3HT refers to poly (3-hexylthiophene), EMIM:TFSI refers to 1-ethyl-3-methylimidazolium bis(trifluoromethylsulfonyl)imide, PVD refers to physical vapor deposition, RIE refers to reactive ion etching, and UV refers to ultraviolet.

**Supplementary Table T2. Estimated read time and read energy consumption for various EGTs^3-20^.**


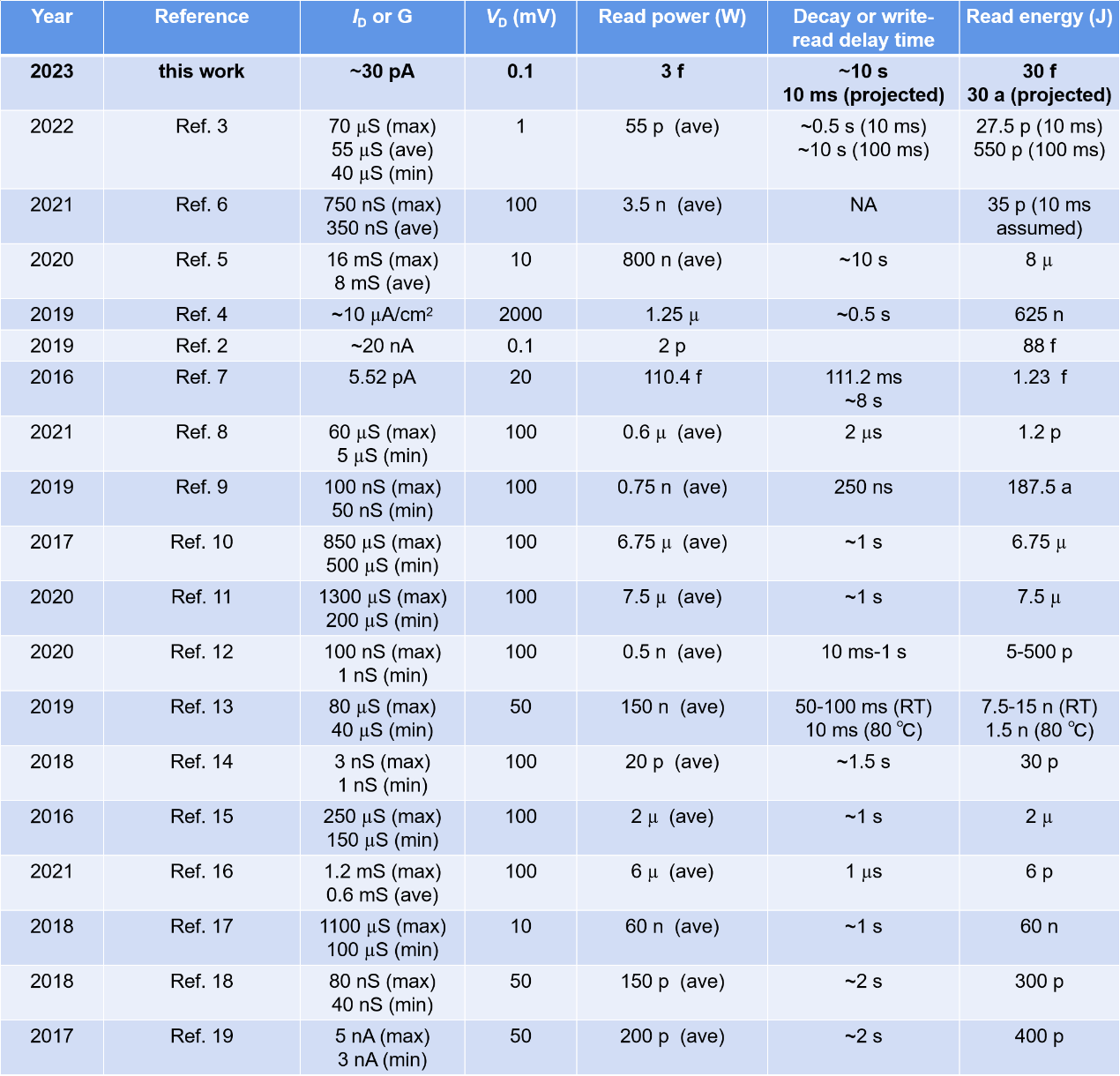


**Supplementary Table T3. Comparison among various EGTs in terms of write energy^3-20^.**

**
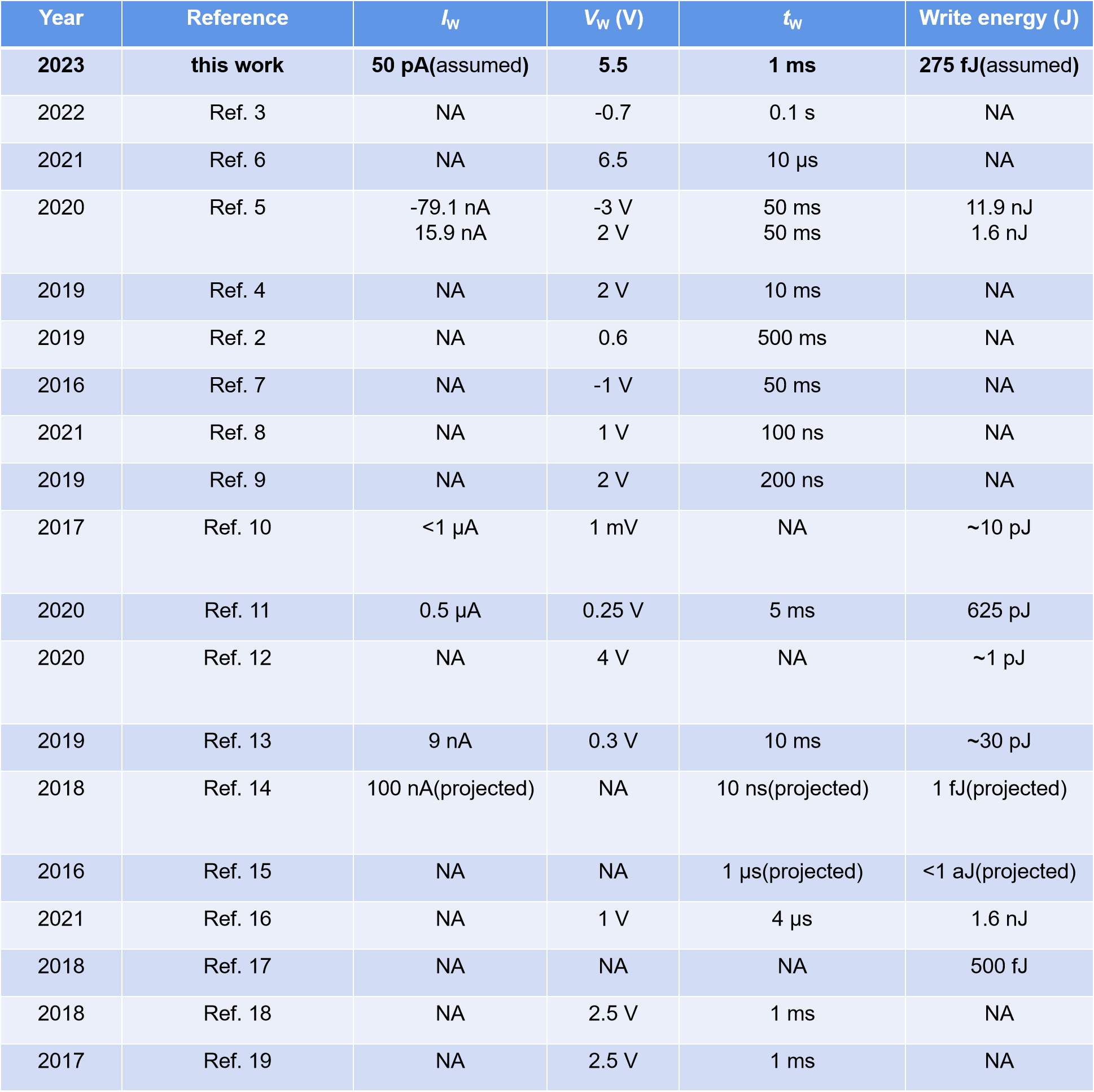
**

**Supplementary Note 1**

**Discussion on the Use of Write-Read Delay of LTM EGTs to Calculate Their Read Energy Consumption**

In this section, we discuss the use of the write-read delay of LTM EGTs as the time to calculate their read energy consumption and the consistency between the decay time of STM EGT and the write-read delay of LTM EGT.

The write-read delay of LTM EGTs is caused by the incomplete conversion of charges injected into the gate during the write process, resulting in only a portion of electrolyte ions migrating toward the electrolyte/channel interface crossing the interface and injecting into the channel, while the rest accumulate on the interface. The former stays in the channel after the gate voltage is removed, leading to the LTM component of EGT, while the latter diffuses back into the electrolyte's interior under the built-in electric field and concentration gradient after the gate voltage is removed, resulting in the STM component of EGT.

From the above analysis, we conclude that after the gate voltage pulse excitation, there is a retreat process of electrolyte ions inside the electrolyte of any EGT, which incurs a time cost of t. For LTM EGT, the time t is the write-read delay, while for STM EGT, it is the decay time. Therefore, to ensure a fair comparison, if we use the decay time of the STM EGT to calculate its read energy consumption, we should use the write-read delay of the LTM EGT to calculate its read energy consumption.

**Supplementary Note 2**

**Discussion on the Decay Time of STM EGT**

Similar to the switching speed of EGT, the decay time of EGT after pulses is determined by the transport of electrolyte ions inside the electrolyte and the transport of carriers inside the channel. Therefore, accelerating these two processes will be beneficial to shorten the decay time of EGT. To accelerate the movement of electrolyte ions inside the electrolyte, thinning the electrolyte thickness (shortening the decay distance), using an electrolyte with higher ionic conductivity (speeding up the decay process), heating (speeding up the decay process), and lowering the triggering intensity of the gate pulse (reducing the amount of decaying ions) can all play a role, and thus help reduce the decay time of EGT. To accelerate the transport of carriers in the channel, reducing the channel length (shortening the transport distance) and using channel materials with higher carrier mobility (speeding up the transport process) will play a role, thus shortening the decay time of EGT.

For polymer channels, a fast switching speed of 2.9 µs has been achieved by mixing electrolyte ions with the channel material^21^. Fuller et al. have also implemented µs-level write and read operations, where the write-read delay of LTM EGT is 250 ns^10^. In contrast, metal-oxide-based EGTs have much larger write-read delay or decay time, typically in the range of 10 ms-1 s. This is because electrolyte ions tend to have much smaller ionic mobility in a solid-phase environment than in a liquid-phase environment. Nevertheless, Li et al. improved the write-read delay of their LTM EGT from 50-100 ms at room temperature to 10 ms at 80℃ by heating^14^. Eckel et al. also reduced the decay time of STM EGT from 10 s (100 ms pulse width) to 0.5 s (10 ms pulse width) by reducing the width of the trigger pulse^4^. Li et al. achieved a write-read delay of 10 ms-1 s for LTM EGT based on α-Nb_2_O_5_ channel^13^.

Therefore, considering that there is still room for reducing electrolyte and channel thickness in our V-EGT, together with reducing gate pulse triggering intensity and high-temperature operation, it is possible for our V-EGT to have a decay time of 10 ms in the future.

The strategies proposed for accelerating the decay process of EGTs are also applicable to improving their operation speed partially. That is, if not preclude the possibility of adopting other materials, there are three methods that can be employed: (1) reducing the thickness of the electrolyte, (2) using an electrolyte material with higher ion conductivity, and (3) heating. Therefore, when these methods are adopted, it is foreseeable for V-EGTs to achieve sub-microsecond operation speeds (<1 µs) in the near future.

**Supplementary Note 3**

**Estimation of V-EGT Device Footprint in Array Configuration**

For V-EGT, the effective device area is considered to be the projection area of the core region on the horizontal plane. Therefore, the area of V-EGT is determined by the electrode width of the source/drain and the gate width. It can be calculated by the product of the source/drain electrode width and the gate width. Accurately, it should be twice the source/drain electrode width multiplied by twice the gate width, considering the spacing of one feature size between adjacent source/drain electrodes and adjacent gates (**Supplementary Fig. 20**).

The device area of V-EGT is limited by the lithographic precision which determines both the source/drain electrode width and the gate width. In our experiments, the typical source/drain electrode width is 10 µm, and the minimum gate width is 2 µm. Therefore, the smallest achieved area of our V-EGT is 10 µm × 2 µm = 20 µm². Within the capability of ultraviolet lithography, the source/drain electrode width can be reduced to a minimum of 1 µm, and the gate width can also be reduced to a minimum of 1 µm. Hence, the minimum device area of our V-EGT can reach 1 µm × 1 µm = 1 µm². Of course, when electron beam lithography is used, the device area of V-EGT can be further reduced.


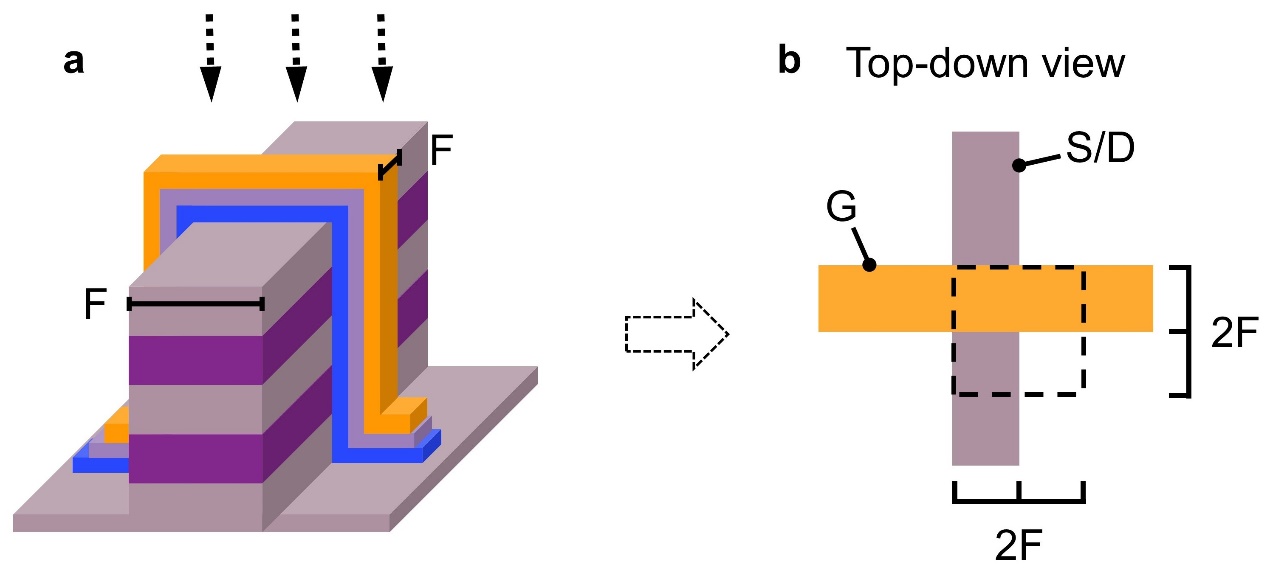


**Supplementary Fig. S20. Calculation of V-EGT device footprint in array configuration. a)** 3D schematics of V-EGT. **b)** Top-down view of **a.**

From a manufacturing process perspective, the etching process still have some influence on the device area of V-EGT, as the width of the source/drain electrode, obtained through etching, is easier to achieve in a larger dimension (e.g., 10 µm) compared to a smaller dimension (e.g., 100 nm). Furthermore, the resistance of TiN source/drain electrodes is also a limiting factor in the device area of V-EGT due to the finite resistivity of TiN. The TiN source/drain electrodes must have a sufficient width to ensure proper device operation. This prerequisite restricts the source/drain electrode width from being arbitrarily small.

In V-EGTs, especially in their array configuration form, mutual interference between adjacent gate electrode stacks has to be considered. There are two forms of potential mutual interference between adjacent gate electrode stacks. The first form occurs when different gate electrodes share the same channel and electrolyte layer (**Fig. S21b**). In this situation, interference between adjacent gate electrode stacks is inevitable, with the severity of the interference increasing as the distance between them decreases. Since electrolyte ions can freely move throughout the electrolyte layer, they can capacitively couple to the same channel, regardless of the distance between different gate electrodes. This capacitive coupling between the gate electrode and the channel is strongly influenced by the distance between them. Consequently, when two gate electrodes are in close proximity, the gate control capability of one electrode over the channel will be significantly affected by the presence of the other electrode. Fortunately, this form of mutual interference is prevented in our case, as different channel/electrolyte/gate stacks are deposited separately (**Fig. S21a**).


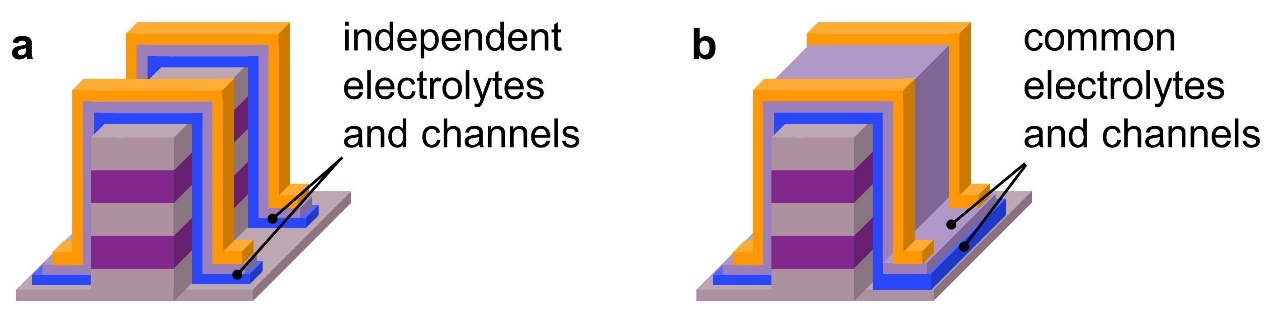


**Supplementary Fig. S21.** Two structures of dual-gate V-EGT. **a)** Dual-gate V-EGT with independent electrolytes and channels. **b)** Dual-gate V-EGT with common electrolytes and channels.

The second type of mutual interference between adjacent gate electrode stacks can be attributed to a parasitic capacitance between nearby conductors. This parasitic capacitance is inherent in almost all 3D-integrated circuits with metal interconnects, and the capacitive coupling between metals or conductors becomes stronger as the distance between them decreases. This effect poses a challenge for miniaturization and high-density array integration. Likewise, when the distance between adjacent gate electrodes becomes sufficiently small, the parasitic capacitance effect of the V-EGT will significantly impact the gate control capability.

**Supplementary Note 4**

**Realizing the Analog Computing Characteristics of Neuron's Coincidence Detection on Dual-Gate V-EGT**

To achieve analog computing characteristics in the coincidence detection of artificial neurons and enable this feature for sound distance recognition, we used a different approach from Das et al. (where coincidence detection can occur at different spatial locations)^22^. We expanded the definition of coincidence detection to include other values of Δt besides 0, thereby realizing analog computing characteristics of coincidence detection.

Thanks to the dual-gate nature of our dual-gate V-EGT, we were able to achieve paired-pulse facilitation (PPF) using two gates. We applied two pulses to two different gates, originally used to achieve regular PPF on the same gate. To distinguish it from single-gate-based PPF, we referred to it as dual-gate PPF. Like regular PPF, dual-gate PPF has the characteristic of nonlinear decay with time interval. By extending the definition of coincidence detection, where coincidence detection can occur at other values of Δt and the PPF index under that Δt serves as the coincidence intensity of the corresponding coincidence detection definition, the coincidence intensity of our device has analog computing characteristics when used for coincidence detection. That is, with the increase of Δt, the corresponding coincidence detection intensity (PPF index) gradually decreases.

**Supplementary Note 5**

**The Principle of Sound Azimuth Recognition Ability of the Dual-Gate V-EGT-Based Neural Network**

Similar to the neural networks constructed by Wang et al.^23^ and He et al.^24^ to emulate sound azimuth recognition in the human brain, we devised the distribution of the synaptic weight matrix to enable our neural network to have sound azimuth recognition ability. Unlike their neural networks, the neural network we constructed also has sound distance recognition ability. Here, we present a general rule for constructing a neural network with sound azimuth recognition ability based on our neural network, although Wang et al. and He et al. have described how to construct such a neural network in detail.

When a sound signal is passed into our neural network, the interaural time difference (ITD) causes a difference in the timing of the left and right ear (sound) sensing neurons receiving the sound signal. Assuming that the sound signal propagates in the neural network in the form of spikes, and the neuron's body integrates information via dendrites, the axon transmits the signal, and the synapse modulates the spike's amplitude (implemented with different synaptic weights). As shown in **Fig. 5e**, the weight distribution (the weight matrix is in the form of a diagonal distribution) leads to opposite spike arrival orders for post-sound information processing neurons 1 and 3. Post neuron 1 receives a small spike first and then a large spike (synaptic weight connections between post neuron 1 and input neuron 1 and 2 were large and small, respectively), while post neuron 3 receives a large spike first and then a small spike (synaptic weight connections between post neuron 3 and input neuron 1 and 2 were small and large, respectively), as shown in **Fig. 5h,i**. Therefore, post neurons 1 and 3 exhibit different peak currents when the second spike passes (**Fig. 5h,i**). Since the ratio between these two current peaks (*I*_post1_/*I*_post3_) has a one-to-one correspondence with ITD, and given the one-to-one correspondence between ITD and sound azimuth, the *I*_post1_/ *I*_post3_ can index only one sound azimuth. Therefore, our neural network can recognize sound azimuth.

**Supplementary Note 6**

**The Principle of Sound Distance Recognition Capability in Our Dual-Gate V-EGT-Based Neural Network**

The human brain is capable of judging the distance of moving objects based on sound intensity. The weaker the sound intensity heard, the farther the sound source is from the human ear; conversely, the stronger the sound intensity, the closer the source is perceived to be. Specifically, sound intensity is inversely proportional to the square of distance. This one-to-one correspondence between sound intensity and object distance allows us to map the relationship between them when either the sound intensity or object distance can be obtained through a measurable physical quantity. Sound intensity can be derived from object distance or object distance can be derived from sound intensity. Therefore, by using a one-to-one correspondence relationship, we can measure a physical quantity to obtain the distance of the object (or sound intensity) and use the relationship between sound intensity and object distance to obtain the sound intensity (or object distance).

In this study, the one-to-one correspondence between coincidence intensity *A*_2_/*A*_1_ and ITD can be used to map the relationship between sound intensity and object distance. The coincidence intensity *A*_2_/*A*_1_ of coincidence detection can be used as the electrical representation of sound intensity. The principle of using the coincidence intensity *A*_2_/*A*_1_ of coincidence detection to identify the distance of sound is as follows: based on the one-to-one correspondence between coincidence intensity *A*_2_/*A*_1_ and ITD, a specific *A*_2_/*A*_1_ corresponds to a unique ITD. According to the relationship between ITD and t (the time it takes for sound to travel from the source to the ear) (**Supplementary Fig. S18**), the unique t and then the distance of the object (by multiplying by the speed of sound) can be indexed from the coincidence intensity *A*_2_/*A*_1_. Therefore, it is feasible to utilize the relationship between coincidence intensity *A*_2_/*A*_1_ and ITD to identify the distance of the sound.

**References**

1. Duan, X.*, et al.* - Novel Vertical Channel-All-Around(CAA) IGZO FETs for $2\mathrm{T}0\mathrm{C}$ DRAM with High Density beyond 4F2 by Monolithic Stacking. **-**, - 10.15.14 (2021).

2. Bu, X.*, et al.* Ion-Gated Transistor: An Enabler for Sensing and Computing Integration. *Advanced Intelligent Systems* **2**, 2000156 (2020).

3. Lenz, J., del Giudice, F., Geisenhof, F.R., Winterer, F. & Weitz, R.T. Vertical, electrolyte-gated organic transistors show continuous operation in the MA cm−2 regime and artificial synaptic behaviour. *Nature Nanotechnology* **14**, 579-585 (2019).

4. Eckel, C., Lenz, J., Melianas, A., Salleo, A. & Weitz, R.T. Nanoscopic Electrolyte-Gated Vertical Organic Transistors with Low Operation Voltage and Five Orders of Magnitude Switching Range for Neuromorphic Systems. *Nano Letters* **22**, 973-978 (2022).

5. Feng, G.*, et al.* A Sub-10 nm Vertical Organic/Inorganic Hybrid Transistor for Pain-Perceptual and Sensitization-Regulated Nociceptor Emulation. *Advanced Materials* **32**, 1906171 (2020).

6. Choi, Y., Oh, S., Qian, C., Park, J.-H. & Cho, J.H. Vertical organic synapse expandable to 3D crossbar array. *Nature Communications* **11**, 4595 (2020).

7. Lee, C., Choi, W., Kwak, M., Kim, S. & Hwang, H. Excellent Synapse Characteristics of 50 nm Vertical Transistor with WO<inf>x</inf> channel for High Density Neuromorphic system. in *2021 Symposium on VLSI Technology* 1-2 (2021).

8. Xu, W., Min, S.-Y., Hwang, H. & Lee, T.-W. Organic core-sheath nanowire artificial synapses with femtojoule energy consumption. *Science Advances* **2**, e1501326.

9. Quill, T.J.*, et al.* Ion Pair Uptake in Ion Gel Devices Based on Organic Mixed Ionic–Electronic Conductors. *Advanced Functional Materials* **31**, 2104301 (2021).

10. Fuller Elliot, J.*, et al.* Parallel programming of an ionic floating-gate memory array for scalable neuromorphic computing. *Science* **364**, 570-574 (2019).

11. van de Burgt, Y.*, et al.* A non-volatile organic electrochemical device as a low-voltage artificial synapse for neuromorphic computing. *Nature Materials* **16**, 414-418 (2017).

12. Yao, X.*, et al.* Protonic solid-state electrochemical synapse for physical neural networks. *Nature Communications* **11**, 3134 (2020).

13. Li, Y.*, et al.* Oxide-Based Electrolyte-Gated Transistors for Spatiotemporal Information Processing. *Advanced Materials* **32**, 2003018 (2020).

14. Li, Y.*, et al.* Low-Voltage, CMOS-Free Synaptic Memory Based on LiXTiO2 Redox Transistors. *ACS Applied Materials & Interfaces* **11**, 38982-38992 (2019).

15. Tang, J.*, et al.* ECRAM as Scalable Synaptic Cell for High-Speed, Low-Power Neuromorphic Computing. in *2018 IEEE International Electron Devices Meeting (IEDM)* 13.11.11-13.11.14 (2018).

16. Fuller, E.J.*, et al.* Li-Ion Synaptic Transistor for Low Power Analog Computing. *Advanced Materials* **29**, 1604310 (2017).

17. Melianas, A.*, et al.* High-Speed Ionic Synaptic Memory Based on 2D Titanium Carbide MXene. *Advanced Functional Materials* **32**, 2109970 (2022).

18. Sharbati, M.T.*, et al.* Low-Power, Electrochemically Tunable Graphene Synapses for Neuromorphic Computing. *Advanced Materials* **30**, 1802353 (2018).

19. Yang, C.-S.*, et al.* All-Solid-State Synaptic Transistor with Ultralow Conductance for Neuromorphic Computing. *Advanced Functional Materials* **28**, 1804170 (2018).

20. Yang, C.S.*, et al.* A Synaptic Transistor based on Quasi-2D Molybdenum Oxide. *Advanced Materials* **29**, 1700906 (2017).

21. Cea, C.*, et al.* Enhancement-mode ion-based transistor as a comprehensive interface and real-time processing unit for in vivo electrophysiology. *Nature Materials* **19**, 679-686 (2020).

22. Das, S., Dodda, A. & Das, S. A biomimetic 2D transistor for audiomorphic computing. *Nature Communications* **10**, 3450 (2019).

23. Wang, W.*, et al.* Learning of spatiotemporal patterns in a spiking neural network with resistive switching synapses. *Science Advances* **4**, eaat4752.

24. He, Y.*, et al.* Spatiotemporal Information Processing Emulated by Multiterminal Neuro-Transistor Networks. *Advanced Materials* **31**, 1900903 (2019).
